# The Midwife1Read at the Buffalo Academy of Medicine, February 28, 1911.

**Published:** 1911-04

**Authors:** P. W. Van Peyma

**Affiliations:** Buffalo, N. Y.; Obstetrician to the Buffalo General Hospital; Clinical Professor of Obstetrics, Medical Department, University of Buffalo; 242 Norwood Avenue


### The Midwife[1]

By P. W. VAN PEYMA, M.D.
Buffalo, N. Y.
Obstetrician to the Buffalo General Hospital
Clinical Professor of Obstetrics, Medical Department, University of Buffalo

HISTORY shows us, as is well known, that the practice of midwifery or obstetrics was from earliest times in the hands of midwives. The medical profession has been wont to recognise Hippocrates as the father of medicine, and since he was the son of a noted midwife, Phainarete, we might, going back one generation, speak of her as the mother of midwifery. This would, however, be untrue to history, as it is well known that midwives practised their vocation centuries preceding even this early date. Not alone in Greece, but in Egypt, India, Japan, Judea, and, in fact, in every country and among all races, from prehistoric times, and through the intervening ages until very recent times, the practice of midwifery was exclusively the province of midwives.

Only very gradually did the practice develop of calling in men to assist in difficult cases. In earliest times these were priests, later medical men, especially surgeons and surgeon barbers. Slowly religious rites, prayers and incantations gave way to surgical procedures; and it is recorded that during the thirteenth century a bishop of Spain, Paulus, performed a cesarian section. But the prejudice against the attendance of men upon women in labor remained strong, and as late as 1522, Dr. Wertt of Hamburg, Germany, having assisted at a labor, dressed as a woman, was burned at the stake. About a century later a Dr. Willoughby of England, being asked by his daughter, a midwife, to assist her in a difficult labor, crawled into the darkened room on his hands and knees.

The history of medicine makes mention of many midwives who attained great renown. Pliny the younger gives a list of midwives who had written on midwifery. Of these, one Aspasia, was the most advanced. About the beginning of the seventeenth century Louise Bourgeois, a graduate of the Hotel Dieu, Paris, was "sage femme" to Marie de Medici, and had the practice of the elite generally. Justinia Sigmundin was midwife to the Court of Brandenburg, and was recognised as an advanced teacher of her time. Mesdames La Chappelle and Boivin, living and prac-

---

1. Read at the Buffalo Academy of Medicine, February 28, 1911.

tising near the beginning of the preceding century, obtained wide celebrity, and very justly so. Both have written works of great practical value, some of which are to be found at the library of the Buffalo Medical College. These and similar works based upon large personal experience are real additions to our store of knowledge, and, for this reason, are far superior to many of the obstetric books of the present day, a large proportion of which are simply compilations, apparently written only to advertise and advantage the compiler.

The midwives in chief of the lying-in hospitals of Germany, and Austria, and other European countries are often women of marked intelligence and ability. Their diagnostic ability not infrequently exceeds that of the medical man in charge.

Of all the countries of Europe England seems from earliest times to have been least inclined to favor midwives. During the eighteenth century the midwives of this country published and distributed pamphlets insisting upon their claims to the domain of obstetric practice. Their efforts met with little favorable recognition. In most of the other European countries, however, they have received full consideration, and special provision has existed for their clinical instruction. In such large obstetric centers as Vienna and Dresden, for example, a portion of the clinical material is devoted exclusively to their instruction.

In the United States the practice of midwives has been almost entirely limited to the foreign population. In this city, for example, chiefly to the Germans, Poles, Italians, and Hungarians.

Little opportunity is given in this country for the practical instruction of midwives, and until recently they were not recognised by law.

In 1885 a law was enacted regulating the practice of midwifery by midwives in Erie County. This law was amended slightly in 1897. The amendments relate to number of members comprising the board of examiners, their terms of office and the amount of fines for transgressions. Similar laws now govern the practice of midwives in other counties of New York state. There is no general state law regulating the practice of midwives. Dr. John H. Pryor is entitled to credit as the original promoter of the bill.

The members comprising the original board were Drs. M. D. Mann, Henry R. Hopkins, Joseph Keene, J. H. Pryor, and P. W. VanPeyma. Since that time, among others, Drs. C. C. Frederick, John Hauenstein, Ludwig Schroeter, Marcel Hartwig, C. E. Congdon have served on the board. At present the members are Drs. John A. Pettit, Charles Jewett, William Thornton, G. Tartaro, M. Kavinoky, L. Kauffman, Frederick Parmenter, C. E. Long, and P. W. Van Peyma.

The following is the text of the original law of 1885:

CHAP. 320.

AN ACT regulating and restraining the practice of midwifery in Erie County by others than legally authorised physicians.

PASSED May 22, 1885, three-fifths being present.

*The People of the State of New York, represented in Senate and Assembly, do enact as follows:*

SECTION 1. On or before the first day of July, eighteen hundred and eighty-five, the county judge of Erie county shall, by an order to be filed in the Erie county clerk's office, appoint a board of examiners in midwifery to consist of five members who shall have been licensed to practise physic and surgery in this state, and thereafter as often as any vacancy shall occur in said board, said county judge shall, by a like order, fill such vacancy.

SEC. 2. Immediately after the filing of said order, said board shall organise by the selection of one of its members as president, and of another as secretary and treasurer, who shall hold their office for one year, and be thereafter annually elected, and shall adopt and have power to adopt and enforce such rules and regulations as are necessary to carry out the purposes and provisions of this act.

SEC. 3. Such examiners shall meet on the first Tuesday of October and April in each year, and on such other days as such board may appoint in the city of Buffalo, after due notice thereof is publicly given, and shall then examine all candidates of the age of twenty-one years and upwards, possessed of good moral character, who shall present themselves to be examined for license to practise midwifery in the county of Erie, and shall, on receipt of ten dollars, issue there certificate to any person so examined who shall be found by them to be qualified, which certificate shall set forth that said board has found the person to whom it is issued qualified to practise midwifery, and shall be recorded by the clerk of the county of Erie in a book to be kept by him for that purpose, All moneys going into the treasury of this board shall be applied to defray the expenses of this board.

SEC. 4. Any person who has received and recorded such certificate shall thereupon be designated a midwife, and authorised and entitled within the county of Erie to practise midwifery in cases of normal labor, and in no others; but such persons shall not in any case of labor use instruments of any kind, nor assist labor by any artificial, forcible or mechanical means, nor perform any version nor attempt to remove adherent placenta, nor administer, prescribe, advise or employ any poisonous or dangerous drug, herb, or medicine, nor attempt the treatment of disease except where the attendance of a physician cannot be speedily procured, and in such cases such person shall at once and in the most speedy way procure the attendance of a physician.

SEC. 5. Said board of examiners shall have power, on proper cause shown, and after hearing the person holding their certificate, to recommend to the county judge of Erie county the revocation of the same, and said judge shall have power to revoke such certificate and license.

SEC. 6. Any person who shall practise or without the attendance of a physician where one can be procured, attend a case of midwifery or obstetrics within the county of Erie, after the thirty-first day of December, eighteen hundred and eighty-five, without being duly authorised so to do under existing laws of this state, or without having received and recorded the certificate provided for by this act, and any person who shall violate any of the provisions of this act shall be guilty of a misdemeanor, and on conviction thereof shall be fined not less than fifty dollars nor more than one hundred dollars, and shall forfeit any certificate theretofore granted under the provisions of this act.

SEC. 7. This act shall take effect immediately.

STATE OF NEW YORK, } ss.:

OFFICE OF THE SECRETARY OF STATE, }

I have compared the preceding with the original law on file in this office, and do hereby certify that the same is a correct transcript therefrom and of the whole of said original law.

JOSEPH B. CARR,
Secretary of State.

At the time of the organisation of the Board of Examiners there were about sixty-five women regularly practising midwifery in the county of Erie. Of these only a very few were located outside the city of Buffalo. A large proportion of these women were graduates of various European schools, and many were women of large practical experience. In addition to these sixty-five, there were many others entirely uneducated, and only occasionally assisting in the cases of neighbors and friends. Many a laundress considered herself competent to officiate as midwife.

The early examinations of the board were not at all severe, it being considered policy not to increase unduly the existing opposition and irritation. At the earlier examinations a stenographer was employed, in order to have legal evidence of the basis of rejection of a candidate. Gradually, however, since that time the standard has been raised. Now and then a candidate from some German, Italian, or other foreign school shows remarkable training. Examinations of candidates include demonstration with the pelvis and manikin. The general appearance of the candidate as to personal cleanliness is noted. The following rules were adopted at the organisation of the board:

Any person applying for examination must furnish satisfactory evidence that she is of good moral character, 21 years of age, or more, and has attended, or assisted in attending, 10 or more cases of labor. The examination will be both written and oral, and the answers must be written or spoken, as the board may direct. Applicants will be examined in English or German, as they may desire. It will be the aim of the board to determine whether a candidate is competent to fulfill the requirements and instructions of the law as stated in Sections 3 and 4, and the character and scope of the examination will be planned with that intention.

The candidate must possess a knowledge of the following essential subjects: The structure of the external and internal parts of the female generative organs and pelvis; the symptoms, mechanism, course and management of natural labor; the symptoms and indications of complicated or abnormal labor, and the emergencies which render it necessary to seek a physician's advice; how to care for the mother and child after the child is born; the hygiene of the sick-room, including cleanliness, etc.; the prevention of disease, and how to avoid infection and contagion.

There are many books which may be consulted, and the board recommends: "A Manual of Midwifery for Midwives and Medical Students," by Fancourt Barnes, M.D., London, and "Lehr-Buch der Hebammenkunst," von Dr. Bernhard Schultze, Leipsig. Further information can be obtained by applying to the secretary, P. W. Van Peyma, M.D., 445 William Street.

Little value is attached to diplomas presented, since occasionally there has existed suspicion of fraudulent deception. During the year 1891 the following circular was issued and sent to licensed midwives. It was published in various languages:

BUFFALO, N. Y., July 1, 1891.

The Board of Examiners in Midwifery for Erie county has already expressed the determination to advise the midwives of the county, from time to time, as to their duties. The wonderful advances, made during recent years, in the prevention of childbed fever in its various forms, makes it imperative that the board should point out to midwives how this is to be accomplished by them in their own practice.

It is practically settled that childbed fever is caused by blood poisoning—the poison being carried into the blood through the open blood vessels of the vagina and uterus, during and after labor. Want of cleanliness on the part of the midwife, especially her hands; on the part of the patient, especially her genitals, and also of her hands, if she touches these parts; on the part of the bedclothes and of the instruments employed, for example, syringes, catheters, etc., are the chief causes of this infection or blood poisoning. To prevent it, then, the patient and the attendant, as well as the bedding and instruments, must be *absolutely clean.* It can be truly said that for every case of childbed fever some one or more persons are *to blame.* The time has come when there need be, and should be, no more childbed fever.

The board, therefore, again calls the attention of midwives to the regulations, which it considers necessary, in the way of cleanliness:

Every midwife, before examining a patient, should see to it that the bedding and the personal clothing of the patient are clean. The patient should receive a bath. The external genitals should be thoroughly cleansed. The vagina should be irrigated. For the irrigation and the bathing of the external genitals, solution No. 1 should be employed.

The hands and forearms of the midwives should be thoroughly scrubbed, for at least five minutes, by means of a brush and warm water and soap. The spaces under and around the finger nails must be especially cleaned, by means of an instrument for that purpose, and by thorough scrubbing these parts with the brush and soap suds. The warm water used for the above purposes should contain Carbolic Acid to the strength of Solution No. 1. Before each of the later examinations, the hands must again be cleaned in the same solution. If a towel is used to dry the hands, this must also be absolutely clean. Before making an examination the finger or fingers, if two are used, must be dipped into vaseline of the strength of No. 2. Immediately after delivery, and twice daily during the first week thereafter, the external genitals should be cleansed, as above directed.

During the lying-in, the patient's clothing and bedding should be kept clean, and changed sufficiently often for this purpose. If a catheter is employed, this should be absolutely clean. If it is of rubber, it should be new, and laid in No. 1 for five minutes, if of metal, it should be placed in boiling water for at least five minutes, and then for at least five minutes more in No. 1. Similar precautions must be exercised with the nozzles of syringes employed.

The large number of cases of inflammation of the eyes in newborn, with the alarming amount of blindness caused thereby, have determined the board to advise the following procedure in all cases. Immediately on the birth of the child, if possible before it has opened its eyes, the lids should be wiped with a soft cloth, moistened in clean lukewarm water. At the time of the first dressing, one or two drops of Solution No. 3 should be dropped into each eye of the newborn. This will result in a slight redness of the eyes for a day or two, and serve as a positive preventive against the disease mentioned. In this connection, attention is called to the appended circular.

Midwives, who neglect either of the above regulations, will be responsible if any trouble occurs as a result of their carelessness and disobedience.

The board desires the names of any persons practising midwifery in the county of Erie without a license. The names must, however, be accompanied by sufficient evidence to prove the case in court.

Address all communications to P. W. Van Peyma, M.D,. secretary.

Solution No. 1 is made by mixing carbolic acid in the proportion of two teaspoonfuls to a pint of water.

Mixture No. 2 is carbolic acid three parts to vaseline one hundred parts. This must be prepared by a druggist, to whom the above must be shown.

Solution No. 3 must be made by druggists, according to the following prescription:

℞ Argenti Nitrat., gram, 1.
Aquae Destil, grams, 50.

M. S. One drop in each eye.
To be kept in dark bottles.

*The People of the State of New York, represented in Senate and Assembly, do enact as follows*:

Section 1. Should any midwife or nurse having charge of an infant in this state, notice that one or both eyes of such infant are inflamed or reddened at any time within two weeks after its birth, it shall be the duty of such midwife or nurse, so having charge of such infant, to report the fact in writing, within six hours, to the health officer or some legally qualified practitioner of medicine, of the city, town or district in which the parents of the infant reside.

Sec. 2. Any failure to comply with the provisions of this act shall be punished by a fine not to exceed one hundred dollars, or imprisonment not to exceed six months, or both.

Sec. 3. This act shall take effect on the first of September, eighteen hundred and ninety.

In the year 1894 a series of lectures was given to midwives in English, German and Polish. Of those practising at the time of the organisation of the board in 1885 only very few are still living, and not more than five or six are known to be still following their profession. During its existence the board has passed and licensed one hundred and seventy. This is about one hundred in addition to those practising in 1885, at the time the board was established. Of course, many candidates failed. Many have been examined repeatedly, and a few of these have eventually passed.

The number of midwives at present in this city is not exactly known. Retirements on account of age, changes of residence, issuance of new licenses, and the like, necessarily result in continual changes. The writer's information, including references to the records of the department of health, leads to the opinion that the number of midwives at present practising in the city of Buffalo is about fifty. It seems certain, then, that the number has not increased with the increase of population. On the contrary, the number is undoubtedly somewhat less than in eighteen hundred and eighty-five.

During the year 1910 there were reported in the city a little over ten thousand births. Upon an examination of one thousand of the certificates on file, assumed to show average conditions, it was found that of this number four hundred and thirty-five were reported by midwives, and five hundred and fifty-three by physicians. If we subtract from the five hundred and fifty-three the comparatively small number born in the various public institutions,—all reported by physicians—it would indicate that practically one-half of the births in private homes are attended by midwives. If the one thousand certificates examined offer a fair average, it would also seem that at least 75 per cent. of the number reported by midwives occur in the practice of not more than ten. It is well known that certain midwives average a case a day. One midwife attended recently 398 cases during twelve months. As to the quality of work done there is, of course, great variety.

The writer's knowledge of the work of midwives extends over a period of thirty-five years. During that time he has had actual knowledge of the practical work of about fifty midwives, this experience totaling several hundred operative cases. The average fee of a midwife is five dollars, which covers the later care of mother and child during the first week at least. The work of midwives can be considered under various heads such as asepsis, diagnostic ability, tendency to exceed legal limitations, induction of abortion, division of fees, etc.

The better midwives understand well and practise conscientiously the asepsis of labor. Many others are more or less wanting in this important matter. Considering the large number of deliveries, occurring often in the worst environment, it is on the whole remarkable that so little sepsis occurs. The midwives have the advantage in this respect over many physicians, whose general practice brings them in contact with infectious diseases, and whose not infrequent hurrying of cases results in severe lacerations, with consequent greater liability to infection of the parturient. The somewhat prevalent belief that sepsis is relatively more common in the practice of midwives than in that of physicians is, at least in part, based on the fact that physicians are often more inclined to diagnose and report infections occurring in the practice of midwives than when occurring in their own cases.

The diagnostic ability of midwives is generally good, and in the cases of many, remarkably excellent. In this respect the average midwife is fully the equal of the average physician. Consultations with fully one hundred physicians in active private practice, and numbering several hundred cases, is the basis upon which this and other statements of comparison are made. The tendency to exceed in their practice the legal limitations is, so far as operative intervention is concerned, much less than formerly. The habit of giving drugs, more or less dangerous, is still occasionally found. The induction of criminal abortion is not chargeable to the vast majority. The division of fees is believed to be not so very uncommon, with the result that not infrequently the choice of physician is not determined by his skill, but rather by considerations more selfish and mercenary. In judging the practice of midwives physicians are apt to hold them up to an ideal standard, and to comment severely on any shortcomings. Judged in this way the practice of obstetrics by physicians in general leaves much to be desired. This fact is quite fully shown in the writer's article on "The Serious Neglect of Scientific Obstetrics."

If we judge the sins of omission and commission of midwives by this comparison it is on the whole favorable. The question is, practically speaking, not so much one of absolute as of relative standard; not so much a question of the ideal as of the best attainable. If we look at the matter fairly and broadly, considering the facts of asepsis, diagnosis and general management, on the one hand, and the question of time involved, artificial deliveries, lacerations, compensation, etc., on the other, the writer is not inclined to believe that the average woman on the east side, now employing a midwife, would on the whole receive better attention and results if she were to employ physicians obtainable for double the fees paid midwives,—to say nothing of the duties of nurse to mother and child, now furnished by the midwife. Another important consideration is a knowledge of the language of the patient. A lying-in institution with beds free, or practically so, seems the only possible solution, if solution seems demanded. And even in such an institution, the active work should be in the hands of men of more than ordinary competency.

In looking back over a period of thirty-five years, making allowance for the fact that the whole science of asepsis has had its practical development during that time, and considering the actual conditions of our foreign population, it seems best to bend our efforts towards improving present conditions, rather than to attempt anything revolutionary, with a view to eliminating the midwife. The remarkably low rate of mortality following careful regulation of the practice of midwives in Italy shows what can be accomplished. It seems probable that in time the midwife will pass away, but this will be as a result of gradual sociologic change, and not something sudden and arbitrary. And when this time shall have come, a just and impartial judgment will give credit for much that was good in the way of painstaking and conscientious care, of days and nights of genuine devotion, of sympathy and cheer, of frequent self-sacrifice to the point of physical endurance. And in the final list of those of whom it can be said that they have been "good and faithful servants," there will appear the name of many a midwife.

242 NORWOOD AVENUE.

